# The impact of asbestos cement pollution in irrigation water on physiological and germination characteristics of *Trifolium pratense*, *Medicago sativa*, and *Solanum lycopersicum* seeds

**DOI:** 10.1038/s41598-025-01011-4

**Published:** 2025-05-09

**Authors:** Gergely Zoltán Macher, András Torma, Dóra Beke

**Affiliations:** 1 Department of Applied Sustainability, Albert Kázmér Faculty of Agricultural and Food Sciences of Széchenyi, István University in Mosonmagyaróvár, Széchenyi István University, Gyor, Hungary; 2 Department of Plant Sciences, Albert Kázmér Faculty of Agricultural and Food Sciences of Széchenyi, István University in Mosonmagyaróvár, Széchenyi István University, Mosonmagyaróvár, Hungary

**Keywords:** Asbestos cement contamination, Asbestos toxicity, Harvested rainwater, Abiotic stress, Plant sciences, Environmental sciences

## Abstract

This paper investigates how plants respond to stress caused by asbestos cement products in irrigation water. It presents a thorough evaluation of the exposure and risk factors for plants, water, and soil when exposed to these materials. The experimental results provide empirical evidence of plant stress responses based on physiological and germination parameters. The research is motivated by concerns about environmental contamination from asbestos cement in irrigation water, which can be toxic to plants and lead to soil pollution, negatively impacting vegetation and soil quality. When exposed to asbestos in water, plants experience toxic stress that can inhibit photosynthesis, nutrient uptake, and germination. Asbestos can also adversely affect cell division and metabolism, risking plant growth, reproduction, and overall health, as well as making them more susceptible to disease and pests under environmental stress. The paper examines the impact on germination and physiological parameters of *Trifolium pratense*, *Medicago sativa*, and *Solanum lycopersicum*, particularly how they were affected by pre-established concentrations of irrigation water mixed with asbestos cement during a controlled germination experiment. The research methodology was developed in the absence of established global practices, standards, and methods, creating an opportunity for further methodological advancement. The findings could serve as a situational analysis for professionals in environmental plant protection and analytical fields.

## Introduction

Asbestos is a collective term for naturally occurring fibrous minerals that were extensively utilized in construction and industry due to their ability to withstand high temperatures and provide insulation. These minerals exhibit durability and cost-effective, but they represent potential health risks when inhaled as airborne particles^1^. Inhalation of asbestos fibres can lead to respiratory illnesses, including asbestosis, lung cancer, and *Mesothelioma malignum*^2^. The presence of asbestos in older structures has been identified as a continuing cause for concern^3^. Asbestos pollution has profound ramifications for environmental wellbeing, especially in soil and water ecosystems.

Asbestos has been shown to significantly affect environmental health, particularly regarding soil and water quality.

The release of asbestos fibres into the environment can contaminate the soil, thereby jeopardizing land-based ecological systems. Soil contamination can contribute to the spread of asbestos through wind erosion or surface runoff, leading to the dispersal of fibres into new areas^4–6^. The enduring nature and resilience of asbestos in the surrounding areas require comprehensive supervision, correction, and precautionary actions to reduce additional environmental and public health consequences^7^.

Asbestos cement products, especially roofing materials, are a major source of asbestos contamination in the environment due to their gradual deterioration. Asbestos cement products, particularly asbestos cement roofing, were widely used due to their durability and resistance to fire and weather. These products consist of a mixture of cement and asbestos fibres, providing structural strength. However, over time, these roofs become increasingly vulnerable to erosion and degradation, leading to the release of asbestos fibres into the environment. Factors such as age, weather conditions, and physical damage can exacerbate this erosion^8^. The deterioration and wearing away of asbestos cement roof materials result in the release of sizable amounts of harmful asbestos fibres into the nearby surroundings. These fibres can endure for extended periods due to their strong resistance to natural degradation processes^9^. Weathering mechanisms like freeze-thaw cycles, acid rain, and ultraviolet radiation expedite the decomposition of these substances, leading to a higher rate of fibre release^10–12^. These fibrous materials can be transported by the wind and distributed in soil or adjacent water sources^13^. Deteriorating roofing can release asbestos fibres, which may contaminate the nearby soil and water, thereby posing serious health risks to human and animal populations^14,15^. Rainwater collected from these roofs has the risk of being contaminated with asbestos fibres, making it unsuitable for drinking or irrigation and possibly causing pollution in local groundwater sources^16–18^. Additionally, the asbestos fibres may settle on plants and vegetation, potentially being incorporated into the food web if these plants are consumed by livestock or wildlife^19^.

Chrysotile asbestos, a component of cement roofing, can be released into water as fibres when the cement materials deteriorate. This release is mainly caused by weathering and erosion processes, during which rainwater or surface runoff carries the asbestos fibres away from their original location^17^. In water, chrysotile asbestos is less durable than other forms, but it can still persist and remain suspended for significant periods due to its fibrous nature^9^. Asbestos fibres that are released into the environment not only present risks to human health and soil quality, but also have direct and indirect impacts on plant physiology and growth.

Investigations into the impacts of asbestos on plants, especially those cultivated for gardens and agriculture, have uncovered multiple effects. Exposure to asbestos fibres can inhibit seed germination, likely due to the abrasive nature of the fibres disrupting seed coats and impeding water uptake^17^. Plants that do germinate may display stunted shoot and root growth because of fibrous asbestos inhibiting the development of root systems, affecting nutrient and water absorption^9^. The number of leaves a plant produces may also be reduced, likely due to impaired photosynthesis and overall vitality^20^. Biochemical factors of plants, such as the level of proteins, may be negatively impacted by exposure to asbestos. The abrasive properties of asbestos fibres along with their chemical reactions can disturb regular metabolic activities. When plant tissues are exposed to soil contaminated with asbestos, the protein content frequently diminishes due to the inhibition of crucial nutrient uptake by the fibres, leading to physiological strain^21^. The presence of asbestos fibres in soil also correlates with reduced chlorophyll content, which can impair photosynthesis and lead to reduced plant vigor and yield^20^. Furthermore, root development can be significantly impacted, as fibres physically obstruct root elongation or chemically disrupt normal root function^22^. Additionally, oxidative stress caused by asbestos exposure can lead to the generation of reactive oxygen species (abbreviation: ROS), which can damage cellular components, further reducing biochemical functions like protein synthesis and leaf growth^23^. Exposure to asbestos poses a distinct challenge to plant life, emerging as a novel environmental element in modern times. The gritty and fibrous quality of asbestos has the potential to disturb cellular arrangements, leading to physical harm in root and leaf tissues. This dual impact results in decreased nutrient absorption, compromised protein production, and overall inhibited growth, culminating in a comprehensive reaction to this stressor^21^. Runoff from deteriorating asbestos cement products can mobilize fibres into collected rainwater, making it a potential hazard when used for irrigation or other purposes^17^. Thus, understanding and mitigating the impact of asbestos on plant stress responses is critical for safeguarding agricultural productivity and ecosystem health in a changing climate. Given these environmental and biological impacts, it is crucial to further investigate how asbestos contamination influences plant stress responses and broader ecological dynamics.

Table 1 presents a comprehensive summary of the relevant literature necessary for establishing the research background of this paper. This summary aims to delineate the existing body of knowledge regarding the effects of chrysotile asbestos on vegetation, thereby clarifying the novelty of the current research. By consolidating previous studies, this overview identifies gaps in the literature and emphasizes the significance of the present investigation. Such an approach ensures that the findings of this research are situated within the broader academic discourse, illustrating how it contributes to advancing the understanding of the relationships between environmental contaminants and plant life. Ultimately, this summary underscores the innovative aspects of the paper, positioning it as an important contribution to the field.

**Table 1:** Summary of the literature background on the effects of Chrysotile asbestos on vegetation.

Author(s) (year)	Title	Findings	Reference
Trivedi A. K., Musthapa A. M. S., Ansari F. A., Rahman Q. (2004)	Environmental contamination of chrysotile asbestos and its toxic effects on growth and physiological and biochemical parameters of *Lemna gibba*	The paper was conducted to assess the toxicity of chrysotile asbestos, a type of asbestos, on Lemna gibba, a water-loving aquatic plant. The research found that asbestos concentrations were present in water, sediment, and aquatic plant samples near an asbestos cement plant. In laboratory experiments, L. gibba plants were exposed to two concentrations of chrysotile asbestos twice weekly for 28 days. The paper evaluated the effects of this exposure on various growth, physiological, and biochemical parameters. The results showed that chrysotile asbestos exposure had inhibitory effects on shoot number, root length, and biomass of the plants. Additionally, it led to changes in chlorophyll, carotenoids, total free sugars, starch, and protein content. On the other hand, electrolyte efflux, lipid peroxidation, cellular hydrogen peroxide, catalase, and superoxide dismutase activity increased in a dose- and time-dependent manner. The findings indicate that chrysotile asbestos causes oxidative stress and phytotoxicity.	^19^
Trivedi A. K., Musthapa A. M. S., Ansari F. A. (2007)	Environmental contamination of chrysotile asbestos and its toxic effects on antioxidative system of *Lemna gibba*	The paper was conducted to evaluate the toxicity of chrysotile asbestos on duckweed plants. Asbestos was found in plant samples near an asbestos cement factory, and laboratory experiments were carried out using different concentrations of chrysotile asbestos to assess changes in antioxidant systems in the plants. The paper measured the levels of glutathione and ascorbate, which are known biomarkers of chrysotile contamination. The results showed that exposure to chrysotile asbestos led to a decrease in total and reduced glutathione, and an increase in oxidized glutathione and reduced/oxidized glutathione ratios. Additionally, there was an increase in the size of the ascorbate pool and in the levels of reduced and oxidized ascorbate, along with a decrease in the reduced/oxidized ascorbate ratio. These changes in antioxidant levels can be considered as indicators of exposure to unsafe environments, as glutathione and ascorbate play a crucial role in combating oxidative stress caused by environmental factors.	^24^
Trivedi A. K., Ahmad I. (2011)	Effects of Chrysotile Asbestos Contaminated Soil on Crop Plants	The paper reports on the impact of the growing vegetation near an asbestoscement factory on chrysotile asbestos. Soil samples collected from locations close to the factory exhibited higher concentrations of asbestos fibres compared to those taken from more distant sites. However, other soil characteristics, such as organic carbon, nitrogen, phosphorus, potassium, electrical conductivity, and pH, were similar across all samples. The food crops used in the experiment, including wheat, peas, and mustard, displayed significant germination issues in soil contaminated with chrysotile asbestos fibres. The height of the exposed plants, root length, biomass, as well as chlorophyll and protein content were also reduced. This paper highlights the detrimental effects of chrysotile asbestos on the surrounding vegetation.	^21^
Trivedi A. K., Ahmad I. (2013)	Impact of chrysotile asbestos contaminated soil on foliar nutrient status of plants	The toxic effects of asbestos on humans are well documented, but little information is available on its effects on plants. In this paper, asbestos contamination was monitored in soil around a cement factory at different distances from the factory. More asbestos contamination was found in the soil near the factory. These soils were analysed for different parameters (organic carbon, EC, pH, macro- and microelements). As the asbestos contamination increased, the levels of magnesium, iron and manganese showed a slight increase, which was accompanied by a decrease in the availability of other nutrients. Wheat, pea and mustard seeds were planted in asbestos-contaminated and control soils. Reduced nutrient content was observed in the leaves of plants grown in asbestos-contaminated soil. The paper reports on the effect of the foliar nutrient status of plants grown near an asbestos cement plant.	^25^
Trivedi A. K., Ahmad I. (2013)	Genotoxicity of chrysotile asbestos on Allium cepa L. meristematic root tip cells	The genotoxic and mutagenic effects of chrysotile asbestos on animals and humans are well-documented, but little is known about its impact on plants. To address this, researchers conducted a paper using Allium cepa L. bulbs exposed to different concentrations of chrysotile asbestos. They observed changes in cytogenetic parameters over a period of 96 h. The paper found that the progression of the prophase stage in exposed plants was slower compared to control plants. The number of cells in metaphase increased in exposed plants, while it decreased in control plants. Furthermore, the number of cells in anaphase and telophase stages decreased in exposed plants over time. The mitotic index also decreased in a time- and concentration-dependent manner in exposed plants. Additionally, exposed plants showed an increase in interphase nuclei, spindle abnormalities, chromosome sticking, and micronucleus formation. These findings suggest that chrysotile asbestos exhibits genotoxicity in plants.	^26^
Saleem K., Asghar M. A., Saleem M. H., Raza A., Kocsy G., Iqbal N., Ali B., Albeshr M. F., Bhat E. A. (2022)	Chrysotile-Asbestos-Induced Damage in *Panicum virgatum* and *Phleum pretense* Species and Its Alleviation by Organic-Soil Amendment	In this paper, the hazardous effects of asbestos on the growth and development of switchgrass and timothy grass were investigated, with the aim of finding ways to mitigate its toxicity. Asbestos-contaminated soil samples were collected near a cement factory, and it was found that these soils had increased uptake of heavy metals, such as chromium, manganese, vanadium, arsenic, and barium, which led to reduced plant growth. To counteract this, antioxidant enzymes were activated to maintain redox balance. The soil closest to the cement plant was found to be the most toxic. However, the addition of compost as a biofertilizer significantly reduced the toxic effect of asbestos fibres and reduced metal uptake, improving overall plant growth and development. It was also observed that heavy metal accumulation was higher in the roots of the grass species. In summary, this paper suggests that the investigated grass species can be negatively affected by asbestos contamination, but the addition of compost can help mitigate these effects.	^27^

The novelty and gap-filling nature of this research can be emphasized by noting that previous literature has typically focused on either the contamination of soil samples taken from polluted sites or analysed aquatic environments. This research integrates these two approaches by examining the various aspects of chrysotile asbestos presence in precipitation and its effects on the soil-plant system. The novelty of this research lies in its comprehensive evaluation of plant stress responses to asbestos cement contamination in irrigation water during the early vegetation phases, an area with limited established global practices and standards. Additionally, there is a notable international gap in that most studies concentrate solely on pure asbestos fibres, overlooking the fact that chrysotile’s high surface tension and charge can lead to the formation of complex associations. A key recognition of this paper is that plants do not encounter pure chrysotile but rather chemically and physically altered complexes or composite formations, which influence their bioavailability and toxic effects. Furthermore, in cases of heavy metal contamination, synergistic effects may occur, which are often disregarded. This paper differs from previous work by employing a multi-faceted approach, integrating lab-based germination and growth experiments with advanced analytical techniques to assess asbestos effects on plants. While earlier studies focused on free asbestos fibres in soil and water, this research examines the impact of asbestos cement particles in irrigation water, simulating real-world contamination. The experimental design uses precise dose-response analyses to evaluate physiological and biochemical stress indicators in plants, while statistical modelling enhances the reliability of findings by quantifying the relationship between asbestos exposure and plant responses. The other novelty of the research lies in the fact that it investigates the combined effect of asbestos-cement matrix, a topic that has been studied only minimally in the literature. Moreover, existing research does not adequately address the involvement of horticulture, further highlighting the significance of the current paper. Thus, this research not only sheds new light on the effects of chrysotile asbestos but also provides a more comprehensive understanding of its impacts on soil-plant systems, particularly focusing on horticulture and the complexities of environmental interactions. Despite the scarcity of such studies, this research provides new insights into the environmental risks posed by asbestos contamination, advances methodological approaches, and offers valuable situational analysis for professionals in environmental plant protection and analytical fields.

## Materials and methods

The paper analysed the germination parameters and diversity of three varieties of *Trifolium pretense* (Salino, Rozeta, Altaswede), *Medicago sativa* (Emiliana, Gea, Algonquin) and *Solanum lycopersicum* (Manó, Kecskeméti 549, Mobil). It also evaluated their variability to validate the adverse effects of asbestos cement contaminated irrigation water on plant growth in a controlled experiment using distilled water. The *Trifolium pratens*e and *Medicago sativa* seeds were procured from Pannon-Mag-Agrár Kft., whereas the *Solanum lycopersicum* seeds were acquired from Garafarm Trade Kft. The results indicated a significant reduction in both the rate and percentage of germination across all *Trifolium pratense*, *Medicago sativa* and *Solanum lycopersicum* varieties. The selected plant species - *Solanum lycopersicum*, *Trifolium pratense*, and *Medicago sativa* - have relevance beyond their agricultural importance, as they can serve as models to address critical environmental and water management concerns. *Solanum lycopersicum* was chosen due to its significance in horticulture and its potential to accumulate contaminants when irrigated with water from aging asbestos cement channels, which poses risks to food safety. *Trifolium pratense* is particularly pertinent in the context of urban green spaces, where the use of greywater for irrigation is an emerging practice, necessitating further assessment of plant resilience and contaminant uptake. *Medicago sativa*, widely cultivated in arable farming, is crucial for evaluating the risks associated with irrigation infrastructure constructed from asbestos cement materials, which may contribute to pollutant exposure. These plant species, therefore, serve as valuable models for understanding the implications of alternative water sources and irrigation systems on both agricultural and ecological sustainability.

### Conditions and arrangement for germination experiment

Three different varieties of *Trifolium pratense*, *Medicago sativa* and *Solanum lycopersicum* were investigated to assess the effects of stress induced by varying doses. The seeds were sterilized on the surface using 2.0% NaOCl for a duration of 2 min, followed by three rinses with sterile distilled water. Subsequently, batches consisting of 10 seeds each were placed in individual sterile Petri dishes containing a moistened cotton disc. Each treatment dose was represented by five petri dishes for analysis (*N* = 5 × 10). There are a total of 350 test items for each type (*N* = 7 × 50). The germination experiment was conducted under controlled laboratory conditions to ensure reproducibility. The Petri dishes housing the seeds were maintained at a consistent temperature of 22 ± 1 °C, monitored using a digital temperature controller. The system utilized built-in LED illumination optimized for plant growth, providing a full-spectrum light source. The LED setup, powered via an adjustable USB-A to USB-C connection, enabled programmable light cycles with seven-step dimmable intensity settings. The photon flux density at plant level was maintained at a maximum of 50 lm. The ventilation system was designed to ensure appropriate airflow regulation.

### Preparation of sample solutions

During this analysis, asbestos cement products were examined that commonly utilized in Hungary, which contained chrysotile asbestos with an average composition ranging from 8.00 to 10.0%, and a cement content of 90.0–92.0%. It was essential to simultaneously investigate these specific characteristics due to their combined effects on the erosion and deterioration of the product matrix. Solutions at various concentrations (1.00 mg/l, 2.00 mg/l, 5.00 mg/l, 10.0 mg/l, 25.0 mg/l, and 50.0 mg/l) were prepared using doubly distilled water in a laboratory setting for analysis purposes. To prepare the extract, asbestos cement samples were first mechanically ground into fine particles. The resulting powder was then suspended in doubly distilled water at predetermined concentrations and incubated for 24 h at ambient temperature. After the extraction process, the suspensions were filtered through a 0.45 μm filter to remove larger particles while retaining the asbestos fibres and colloidal components. The resulting solutions were stored in sterile glass containers under dark conditions at 4 °C until needed for further analysis. The control group underwent treatment with double-distilled water. Seeds were subjected to their assigned dosage and treatment method during the experiment.

### Germination assessment

Throughout the analysis, we consistently monitored the germination rate, germination time, and the root length and shoot height of plants. Root length was measured from the point of contact with the cotton disc’s surface to the tip of the primary root, while shoot height measurements were taken on day 31 for each sample. These parameters were assessed utilizing a Burg-Wächter Precise PS-72,150 digital caliper (0 mm to 150 mm range) to ensure precise data on development. Each treatment was replicated multiple times, and our findings are reported as mean values ± standard error. The obtained data was examined in terms of percentages. The statistical significance of the results was evaluated using ANOVA analysis performed in Excel, with a significance level threshold of 0.05 to determine if the findings were statistically significant.

## Results

### Results of *Trifolium pratense* analysis

**Fig. 1:**
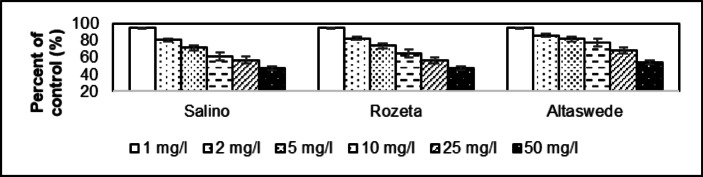
Comparative examination of root length in *Trifolium pratense* cultivars (Salino, Rozeta, and Altaswede) exposed to asbestos-contaminated irrigation water. Significant reductions in root length were observed at both 1 mg/l and 50 mg/l concentrations (*p* ≤ 0.05), with greater inhibition at higher doses. The reduction was most prominent in the 50 mg/l dose, where root length was reduced to 47.6% (Salino), 47.8% (Rozeta), and 54.5% (Altaswede) compared to the control group.

The control germination rate is 88.0% for Salino, 86.0% for Rozeta and 90.0% for Altaswede. Germination of *Trifolium pratense* seeds at 25.0 °C under light and dark conditions averaged 88.0 ± 2.00%. The tested samples showed that already at a dose concentration of 1 mg/l the germination rate was reduced to 82.0% in the case of the Salino, 80.0% for Rozeta and 84.0% for Altaswede. This was reduced to 48.0% (Salino), 46.0% (Rozeta) and 58.0% (Altaswede) at the 50 mg/l dose, and to 54.5% (Salino), 53.5% (Rozeta) and 64.4% (Altaswede) as a percentage of the control group. The average value for the whole group is 93.2 ± 0.16% for 1.00 mg/l and 57.5 ± 6.04% for 50 mg/l at the percentage of control group. The average difference in the effect of the two dose concentrations is −35.7 ± 5.90%. The coefficient of determination of Salino is 0.9840, while 0.9974 for Rozeta and 0.9313 for Altaswede. The dose effect of 1 mg/l resulted in a reduction to 95.2% in root length compared to the control group in Salino (Fig. 1). This rate was 95.7% for Rozeta and 95.5% for Altaswede. The values as a percentage of the control group because of the 50 mg/l dose effect was 47.6% for Salino, 47.8% for Rozeta, and 54.5% for Altaswede. This represented an average of 95.4 ± 0.21% (1.00 mg/l) and 50.0 ± 3.94% (50.0 mg/l). The coefficient of determination of Salino is 0.9791, while 0.9941 for Rozeta and 0.9611 for Altaswede.

**Fig. 2:**
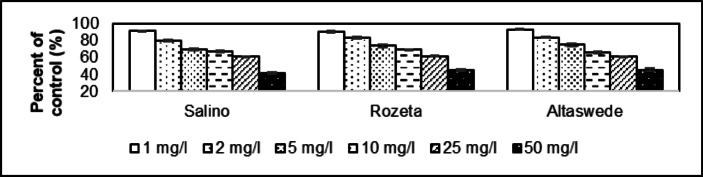
Comparative examination of shoot height in *Trifolium pratense* cultivars (Salino, Rozeta, and Altaswede) exposed to asbestos-contaminated irrigation water. Significant reductions in shoot height were observed at both 1 mg/l and 50 mg/l concentrations (*p* ≤ 0.05), with greater inhibition at higher doses. The reduction was most prominent in the 50 mg/l dose, where shoot height was reduced to 41.3% (Salino), 45.2% (Rozeta), and 45.5% (Altaswede) compared to the control group.

The dose effect of 1 mg/l resulted in a reduction to 91.3% in height of the shoot compared to the control group in Salino (Fig. 2). This value was 90.5% for Rozeta and 93.2% for Altaswede. The average value as a percentage of the control group because of the 50 mg/l dose effect was 41.3% for Salino, 45.2% for Rozeta, and 45.5% for Altaswede. The average of 1.00 mg/l dose affect is 91.7 ± 1.39% and for 50.0 mg/l is 44.0 ± 2.34%. The coefficient of determination of Salino is 0.9439, while 0.9662 for Rozeta and 0.9828 for Altaswede.

### Results of *Medicago sativa* analysis

**Fig. 3:**
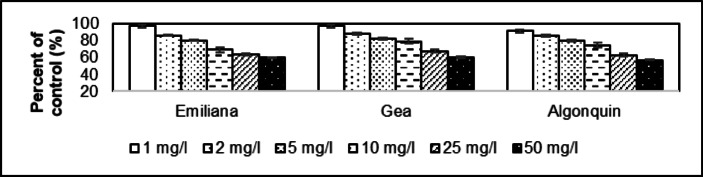
Comparative examination of root length in *Medicago sativa* cultivars (Emiliana, Gea, and Algonquin) exposed to asbestos-contaminated irrigation water. Significant reductions in root length were observed at both 1 mg/l and 50 mg/l concentrations (*p* ≤ 0.05), with greater inhibition at higher doses. The reduction was most prominent in the 50 mg/l dose, where root length was reduced to 59.7% (Emiliana), 60.3% (Gea), and 57.1% (Algonquin) compared to the control group.

The control germination rate is 92.0% for Emiliana, 94.0% for Gea and 90.0% for Algonquin. Germination of *Medicago sativa* seeds at 25.0 °C under light and dark conditions averaged 92.0 ± 2.00%. The tested samples showed that already at a dose concentration of 1 mg/l the germination rate was reduced to 88.0% in the case of the Emiliana and Gea, while it was 86.0% for Algonquin. This was reduced to 54.0% (Emiliana), 50.0% (Gea) and 52.0% (Algonquin) at the 50 mg/l dose, and to 58.7% (Emiliana), 53.2% (Gea) and 57.8% (Algonquin) as a percentage of the control group. The average value for the whole group is 94.9 ± 1.15% for 1.00 mg/l and 56.6 ± 2.95% for 50 mg/l at the percentage of control group. The average difference in the effect of the two dose concentrations is −38.4 ± 1.81%. The coefficient of determination of Emiliana is 0.9827, while 0.9842 for Gea and 0.9754 for Algonquin. The dose effect of 1 mg/l resulted in a reduction to 97.2% in root length compared to the control group in Emiliana (Fig. 3). This rate was 97.1% for Gea and 91.4% for Algonquin. The values as a percentage of the control group because of the 50 mg/l dose effect was 59.7% for Emiliana, 60.3% for Gea, and 57.1% for Algonquin. This represented an average of 95.2 ± 3.30% (1.00 mg/l) and 59.1 ± 1.68% (50.0 mg/l). The coefficient of determination of Emiliana is 0.9781, while 0.9788 for Gea and 0.9844 for Algonquin.

**Fig. 4:**
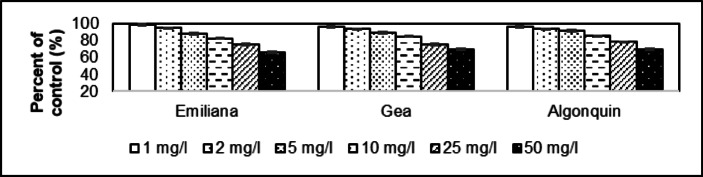
Comparative examination of shoot height in *Medicago sativa* cultivars (Emiliana, Gea, and Algonquin) exposed to asbestos-contaminated irrigation water. Significant reductions in shoot height were observed at both 1 mg/l and 50 mg/l concentrations (*p* ≤ 0.05), with greater inhibition at higher doses. The reduction was most prominent in the 50 mg/l dose, where shoot height was reduced to 65.9% (Emiliana), 69.1% (Gea), and 69.9% (Algonquin) compared to the control group.

The dose effect of 1 mg/l resulted in a reduction to 98.8% in height of the shoot compared to the control group in Emiliana (Fig. 4). This value was 96.3% for Gea and 96.4% for Algonquin. The average value as a percentage of the control group because of the 50 mg/l dose effect was 65.9% for Emiliana, 69.1% for Gea, and 69.9% for Algonquin. The average of 1.00 mg/l dose affect is 97.2 ± 1.41% and for 50.0 mg/l is 68.3 ± 2.14%. The coefficient of determination of Emiliana is 0.9818, while 0.9604 for Gea and 0.9429 for Algonquin.

### Results of *Solanum lycopersicum* analysis

**Fig. 5:**
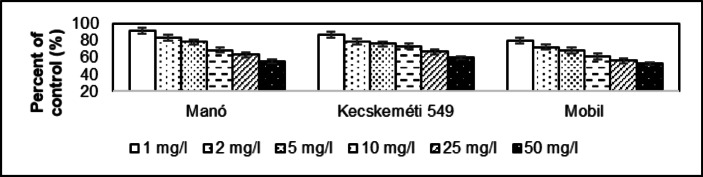
Comparative examination of root length in *Solanum lycopersicum* cultivars (Manó, Kecskeméti 549, and Mobil) exposed to asbestos-contaminated irrigation water. Significant reductions in root length were observed at both 1 mg/l and 50 mg/l concentrations (*p* ≤ 0.05), with greater inhibition at higher doses. The reduction was most prominent in the 50 mg/l dose, where root length was reduced to 55.4% (Manó), 59.7% (Kecskeméti 549), and 52.6% (Mobil) compared to the control group.

The control germination rate is 92.0% for Manó and 90.0% for Kecskeméti 549 and Mobil. Germination of *Solanum lycopersicum* seeds at 25.0 °C under light and dark conditions averaged 90.7 ± 1.15%. The tested samples showed that already at a dose concentration of 1 mg/l the germination rate was reduced to 88.0% in the case of the Manó and Kecskeméti 549, while 86.0% for Mobil. This was reduced to 58.0% (Manó), 60.0% (Kecskeméti 549) and 56.0% (Mobil) at the 50 mg/l dose, and to 63.0% (Manó), 66.7% (Kecskeméti 549) and 62.2% (Mobil) as a percentage of the control group. The average value for the whole group is 96.3 ± 1.26% for 1.00 mg/l and 64.0 ± 2.36% for 50 mg/l at the percentage of control group. The average difference in the effect of the two dose concentrations is −32.4 ± 1.13%. The coefficient of determination of Manó is 0.9909, while 0.9823 for Kecskeméti 549 and 0.9916 for Mobil. The dose effect of 1 mg/l resulted in a reduction to 91.9% in root length compared to the control group in Manó (Fig. 5). This rate was 87.5% for Kecskeméti 549 and 80.3% for Mobil. The values as a percentage of the control group because of the 50 mg/l dose effect was 55.4% for Manó, 59.7% for Kecskeméti 549, and 52.6% for Mobil. This represented an average of 86.6 ± 5.87% (1.00 mg/l) and 55.9 ± 3.57% (50.0 mg/l). The coefficient of determination of Manó is 0.9961, while 0.9710 for Kecskeméti 549 and 0.9908 for Mobil.

**Fig. 6:**
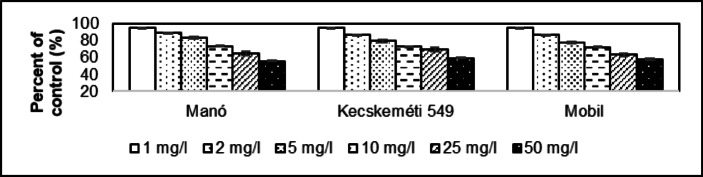
Comparative examination of shoot height in *Solanum lycopersicum* cultivars (Manó, Kecskeméti 549, and Mobil) exposed to asbestos-contaminated irrigation water. Significant reductions in shoot height were observed at both 1 mg/l and 50 mg/l concentrations (*p* ≤ 0.05), with greater inhibition at higher doses. The reduction was most prominent in the 50 mg/l dose, where shoot height was reduced to 56.0% (Manó), 59.6% (Kecskeméti 549), and 57.8% (Mobil) compared to the control group.

The dose effect of 1 mg/l resulted in a reduction to 95.6% in height of the shoot compared to the control group in Manó (Fig. 6). This value was 95,5% for Kecskeméti 549 and 95,6% for Mobil. The average value as a percentage of the control group because of the 50 mg/l dose effect was 56.0% for Manó, 59.6% for Kecskeméti 549 and 57.8% for Mobil. The average of 1.00 mg/l dose affect is 95.6 ± 0.05% and for 50.0 mg/l is 57.8 ± 1.75%. The coefficient of determination of Manó is 0.9916, 0.9863 for Kecskeméti 549 and 0.9941 for Mobil.

## Discussion

Previous research^28^has shown that leguminous plants respond to chemical stressors in a dose-dependent manner^29,30^. Our findings confirm this trend, as *Trifolium pratense* exhibited significantly lower germination rates at higher doses.

This sensitivity is critical to understanding the ecological implications of chemical exposure, as legumes play a vital role in soil health and fertility due to their ability to fix atmospheric nitrogen^31,32^. The low germination rates at higher doses indicate that *Trifolium pratense* has a relatively low chemical tolerance. This may negatively impact its population dynamics and the stability of the surrounding ecosystem.

Many studies have shown that leguminous plants suffer reduced growth and reproductive success when exposed to pollutants^33,34^. Our findings support this, highlighting the potential risks to their ecological role and agricultural importance. The results are not only striking in the case of *Trifolium pratense*, but also evident in the two other receptors, which similarly responded to the effects of chemical stressors. Throughout the studies, we observed a significant decrease in germination rates for the other two receptors as well, reinforcing the notion that chemical pollution has widespread effects on various plant species. These findings suggest that the examined chemical substances exert influence not only on specific plants but also on surrounding ecosystems. The diversity of reactions triggered by chemical stressors may yield important insights into maintaining ecological balance and underscores the necessity for further research to achieve a deeper understanding of this phenomenon.

It is crucial to consider that the observed effects may be influenced by the concurrent action of multiple stressors present in the environment. For instance, the leaching of other toxic substances, such as heavy metals or construction-related contaminants, from polluted sites could also contribute to the observed responses. Investigating these interactions and isolating the effects of a single chemical stressor would necessitate further research into the potential cumulative impacts of various pollutants.

Asbestos exposure can disrupt key biological processes in plants. This disruption can lead to abnormal growth patterns, especially in the root and shoot systems^35^. Additionally, asbestos exposure may impair nutrient uptake by damaging root membranes or inducing oxidative stress, which compromises cellular transport proteins involved in nutrient absorption^36–38^. These cellular disturbances contribute to significant reductions in germination rates, root growth, and shoot height observed in the study, as plants exposed to high asbestos concentrations exhibit stunted development and decreased ability to absorb essential nutrients^39,40^.

The effects of asbestos on plant growth can also be linked to oxidative stress pathways. When exposed to toxic substances like asbestos, plants often experience an increase in reactive oxygen species, which can damage lipid membranes, proteins, and DNA^41,42^. This oxidative stress disrupts normal cell functioning, leading to impaired metabolic processes and growth inhibition. Specifically, asbestos exposure may trigger an imbalance between antioxidants and ROS, overwhelming the plant’s defense systems. As a result, plants may exhibit stunted growth, reduced germination rates, and inhibited root elongation, as observed in our study^43^. These effects align with previous research indicating that oxidative stress is a major factor in plant response to chemical pollutants, as ROS accumulation can interfere with nutrient uptake and cellular respiration^44^. Therefore, oxidative stress pathways may play a central role in the plant’s inability to tolerate high concentrations of asbestos, contributing to the observed toxic effects on plant development.

Similarly, the significant reduction in root growth at 50 mg/l aligns with other research^45^showing inhibition of root elongation by comparable phytotoxin concentrations^46–48^. The decrease in shoot height under higher dose conditions is supported by findings^49^documenting growth inhibition under stressful environmental toxin conditions, involving hormonal imbalances and nutritional uptake disruptions^50,51^. These dose-dependent inhibitory effects are consistent with broader plant toxicology results^52^where higher doses impact plant physiological and metabolic processes^53–57^.

In light of these complexities, it is essential to consider potential confounding factors, such as variations in nutrient availability, water quality, or climatic conditions, which may further complicate the interpretation of dose-response relationships. These factors could independently influence plant health, irrespective of the chemical stressors under investigation. Therefore, it is recommended that future studies either control for these variables or conduct research in a more controlled experimental setup to more accurately isolate the effects of the pollutants.

These findings suggest important implications for environmental policies and strategies to mitigate pollution. The observed phytotoxic effects emphasize the necessity of regulating contaminant levels in agricultural and natural environments to safeguard plant biodiversity and ecosystem stability^58,59^. Implementing more stringent guidelines on permissible pollutant concentrations in irrigation water and soil can help minimize the adverse impacts of chemical stressors^60^. Additionally, the results highlight the importance of adopting sustainable agricultural practices, such as phytoremediation and buffer zone management, to alleviate the impact of contaminants on vegetation^61,62^. These approaches align with broader environmental policies focused on enhancing soil health, maintaining biodiversity, and ensuring food security^63^.

Such findings highlight the importance of considering dose-response relationships in assessing the risks posed by chemical stressors, particularly in environments where these species are cultivated or naturally occur. Further research is warranted to explore the mechanisms behind this sensitivity and to evaluate the long-term implications for plant communities and agricultural practices. The wider environmental ramifications of these findings also highlight the need for more robust mitigation strategies and policy interventions^64,65^. Given the demonstrable adverse impacts of chemical contamination, including asbestos pollution, on plant growth and ecosystem resilience, regulatory frameworks should prioritize monitoring and remediation initiatives^66^. This underscores the imperative of integrating scientific evidence into policy development to mitigate environmental degradation and safeguard biodiversity^5^. The findings highlight the need to address the real-world challenges for farming in areas with asbestos contamination. The negative impacts on plant growth and germination show the risks to crops grown in polluted soils or irrigated with asbestos-tainted water^67^. Farmers and land managers in affected regions should explore alternative water sources, soil remediation methods, and more resilient plant species^68^. This is especially crucial for sustainable agriculture in historically industrial or asbestos-exposed areas^14^. Further research should focus on practical ways to mitigate these impacts while maintaining agricultural productivity and food safety.

## Conclusions

The paper’s results indicate a relationship between the dosage and its effect on seed germination, root length, and shoot height in *Trifolium pratense*, *Medicago sativa*, and *Solanum lycopersicum*. At 1 mg/l concentration, there was minimal impact on germination and growth similar to the control conditions. However, at the higher dosage of 50 mg/l, all parameters showed significant decreases. This significant decrease demonstrates how vulnerable seed germination and seedling development are to increased chemical exposure. The high determination coefficients for germination, root length, and shoot height underline the strong connection between higher dosages leading to negative effects. Despite consistent patterns observed across different cultivars indicates that inherent genetic factors affect resilience. These findings stress the need to understand specific responses of each species or cultivar for better agricultural management aiming at protecting crop yield as well as environmental health. There is also a necessity for further research into identifying both physiological and molecular mechanisms behind these responses which will help enhance crop resilience strategies.

## Data Availability

The datasets generated during and analysed during the current paper are not publicly available but are available from the corresponding author on reasonable request.
